# Revolutionizing organic synthesis through green chemistry: metal-free, bio-based, and microwave-assisted methods

**DOI:** 10.3389/fchem.2025.1656935

**Published:** 2025-08-04

**Authors:** Sayani Banerjee, Sakthi Periyasamy, Kathiravan Muthukumaradoss, Priya Deivasigamani, Venkatesan Saravanan

**Affiliations:** ^1^ Department of Pharmaceutical Chemistry, SRM College of Pharmacy, SRMIST, Chengalpattu, Tamil Nadu, India; ^2^ Department of Pharmaceutical Chemistry, Saveetha College of Pharmacy, Saveetha Institute of Medical and Technical Sciences, Saveetha University, Chennai, India

**Keywords:** green chemistry, sustainable synthesis, eco-friendly catalysis, ionic liquids, microwave-assisted

## Abstract

The growing emphasis on sustainable development has propelled green chemistry into a vital framework for designing environmentally benign chemical processes. This review highlights recent advancements in green methodologies for organic synthesis, emphasizing strategies that reduce the use of hazardous reagents and solvents while enhancing efficiency and atom economy. Key approaches include solvent-free reactions; the use of water and ionic liquids as green solvents; biocatalysis employing plant extracts and natural acids; and microwave-assisted synthesis. Notable progress in metal-free oxidative coupling—particularly for synthesizing 2-aminobenzoxazoles, imidazoles, pyrazoles, and Schiff bases—demonstrates the shift away from traditional transition-metal catalysis toward safer alternatives. In addition, innovative transformations using natural catalysts such as pineapple juice and onion peel, microbial biotransformations, and bio-based solvents like eucalyptol and ethyl lactate illustrate the expansive potential of green chemistry. Techniques such as photocatalysis and phase-transfer catalysis further exemplify energy-efficient and selective processes. Collectively, these methods offer high yields, shorter reaction times, and significant environmental benefits. This review underscores the practicality and promise of green chemistry in advancing sustainable organic synthesis, particularly within the pharmaceutical and fine chemical industries.

## Introduction

The concept of green chemistry has gained significant attention in recent years as a sustainable approach to the synthesis of organic compounds. This growing discipline promotes the practical application of principles aimed at reducing the use and generation of hazardous substances, thereby minimizing the environmental impact of chemical processes. Key strategies include the use of solvent-free or environmentally benign solvents—preferably water—alternative reaction media, one-pot syntheses, multicomponent reactions, continuous flow processes, and process intensification. These methods contribute to improved atom economy and reduced waste generation, aligning with the core goals of green chemistry (Kovacs et al., 2019). Green synthesis can be achieved by maximizing resource efficiency, reducing hazards and pollution, and designing the entire life cycle of an active pharmaceutical ingredient (API) with sustainability in mind. Accordingly, this review focuses on organic synthesis approaches that employ greener solvents such as non-hazardous alternatives—including ionic liquids—along with the use of plant extracts, fresh fruit juices, metal-free conditions, and water as eco-friendly media. The aim is to offer chemists practical insights into conducting organic reactions in safer, more environmentally sustainable ways (Constable, n.d.; Ratti, 2020; Ciriminna et al., 2024).

Over the past decade, many have pioneered direct C-H amination reactions that display improved atom efficiency. However, most of the methods require transition metals like copper, silver, manganese, iron, or cobalt to work. The toxicity and cost of these metals may limit the practical applications of direct C-H amination. Recently metal-free Catalysed oxidative coupling reactions have made rapid progress to overcome the drawbacks of transition metal catalysis. The discovery of green and sustainable oxidative C-H amination of benzoxazoles under metal-free conditions would be very valuable. Hypervalent iodine compounds have garnered much attention in organic synthesis as versatile and potent oxidants (Asveld, 2019; Ganesh et al., 2021; Puri et al., 2024; Raj Joshi and Adhikari, 2019; Ratti, 2020; Thirumavalavan et al., 2024; Yuan et al., 2022).

## Synthesis of 2-aminobenzoxazoles

### Using metal-free conditions by green technique

In a notable study (Joseph et al., 2011), employed stoichiometric amounts of PhI(OAc)_2_ to achieve the direct oxidative C–H amination of benzoxazoles. Similarly, Bhanage et al. utilized 2-iodoxybenzoic acid (IBX) for the same transformation. Significant progress has also been made by combining an iodine source with an oxidant to facilitate this reaction. In contrast, Lamani and Prabhu reported a metal-free oxidative amination approach, using molecular iodine as the catalyst and tert-butyl hydroperoxide (TBHP) as the oxidant. Additionally, Nachtsheim’s et al., developed a metal-free method for the oxidative C–H amination of benzoxazoles, employing tetrabutylammonium iodide (TBAI) as a catalyst along with aqueous solutions of H_2_O_2_ or TBHP as co-oxidants at 80°C. In the conventional method, Cu(OAc)_2_ and K_2_CO_3_ are used to catalyze the reaction between o-aminophenol and benzonitrile, yielding approximately 75%. However, the reagents involved in this method are known to pose significant hazards to the skin, eyes, and respiratory system (Zhou et al., 2017).

### Using ionic liquids by the green chemistry technique

**SCHEME 1:**
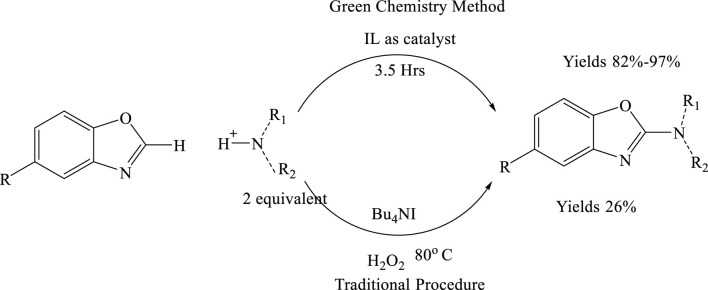
Comparison of traditional and green synthetic approaches for the formation of 2-substituted benzoxazoles.

Ionic liquids (ILs) have garnered significant interest among chemists as green reaction media, owing to their unique chemical and physical properties, such as high thermal stability, negligible vapor pressure, and non-flammability. In recent years, ILs have been increasingly utilized as solvents in transition metal-catalysed C–H activation reactions. However, classical heterocyclic ILs acting as promoters or catalysts in C–H bond activation remains relatively rare. A notable advancement includes the development of an oxidative cross-coupling reaction for C–C bond formation promoted by the ionic liquid 1,3-dibutyl-1H-benzo[d][1,2,3]triazol-3-ium bromide. Shortly thereafter, the first IL-catalysed C–H activation reaction for C–N bond formation was reported. This method employed the heterocyclic ionic liquid 1-butylpyridinium iodide ([BPy]I) as a catalyst, tert-butyl hydroperoxide (TBHP) as the oxidant, and acetic acid as an additive, proceeding efficiently at room temperature (8) (Scheme 1).

Advantages: The traditional synthesis of 2-aminobenzoxazoles has been associated with relatively low yields, limiting its efficiency and scalability. The introduction of ionic liquids (ILs) as reaction media has significantly improved the outcome of this transformation. ILs not only enhance the overall reaction efficiency but also lead to a substantial increase in product yield, now ranging between 82% and 97%. This notable improvement underscores the effectiveness of ionic liquids in optimizing synthetic protocols, offering a more sustainable and practical route for the efficient production of 2-aminobenzoxazoles (Zhou et al., 2017).

## Synthesis of isoeugenol methyl ether (IEME) from eugenol

### By O-methylation

O-Methylation of phenolic compounds is a key transformation in organic synthesis, particularly in the production of fragrance compounds. This reaction involves the conversion of phenols into aryl methyl ethers through the reaction of the hydroxyl group with a methylating agent. While conventional methylating agents such as dimethyl sulfate and methyl halides are effective, their high toxicity and environmental hazards limit their practical use. Dimethyl carbonate (DMC) has emerged as a sustainable and environmentally benign alternative. As a green reagent, DMC can effectively replace hazardous methylating agents in O-methylation reactions. In addition to its methylating ability, DMC serves as a non-toxic solvent, fuel additive, and intermediate in pharmaceutical and chemical industries. For O-methylation of phenols, DMC has been employed in the presence of basic catalysts such as alkali metals, tertiary amines, phosphonium salts, zeolites, alumina, and silica-alumina. The one-step synthesis of isoeugenol methyl ether (IEME) was optimized using various catalysts and phase-transfer catalysts (PTCs). The optimized reaction conditions included a DMC drip rate of 0.09 mL/min, a temperature of 160°C, a duration of 3 h, and a reactant molar ratio of 1:4:0.1:0.1 for eugenol, DMC, catalyst, and PTC, respectively (Zhang et al., 2024).

### By allylbenzene isomerization

**SCHEME 2:**
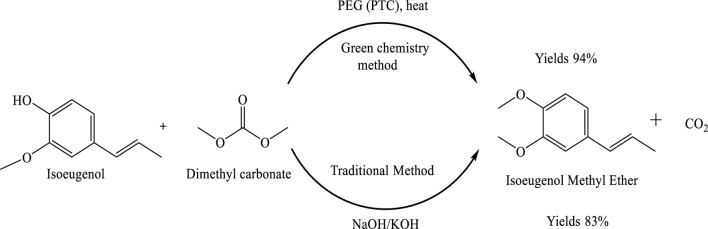
Comparative synthesis of isoeugenol methyl ether using traditional and green chemistry methods. The green method employs dimethyl carbonate (DMC) and polyethylene glycol (PEG) as a phase-transfer catalyst under heat, affording a higher yield (94%) of the desired product. In contrast, the traditional method using strong bases such as NaOH or KOH results in a lower yield (83%).

The isomerization of 2-propenylbenzene to 1-propenylbenzene is an important transformation in various industrial applications. Traditionally, this reaction requires high temperatures and strong bases such as potassium hydroxide (KOH) or sodium hydroxide (NaOH), which pose environmental and safety concerns. The use of phase-transfer catalysts (PTCs), such as polyethylene glycol (PEG), offers a more sustainable and efficient alternative by facilitating reactions between immiscible phases. In this study, a green, one-step synthesis of isoeugenol methyl ether (IEME) was investigated using dimethyl carbonate (DMC) as a methylating agent and PEG as the PTC. The reaction conditions were optimized to develop a safer, more economical, and environmentally friendly process, demonstrating the effectiveness of this approach in both isomerization and O-methylation under mild and sustainable conditions, which is shown in Scheme 2 (Zhang et al., 2024).

## Five-membered aromatic nitrogen heterocycles

### Formation of the pyrrole ring by reactions in PEG

**SCHEME 3:**
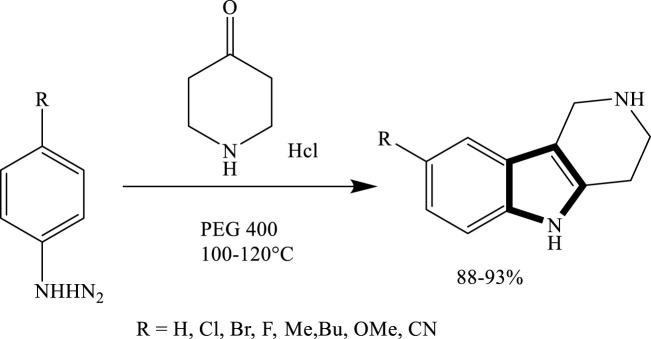
Green synthesis of substituted tetrahydrocarbazoles via the condensation of phenylhydrazine derivatives with 4-piperidone hydrochloride in PEG-400 at 100°C–120°C.

**SCHEME 4:**
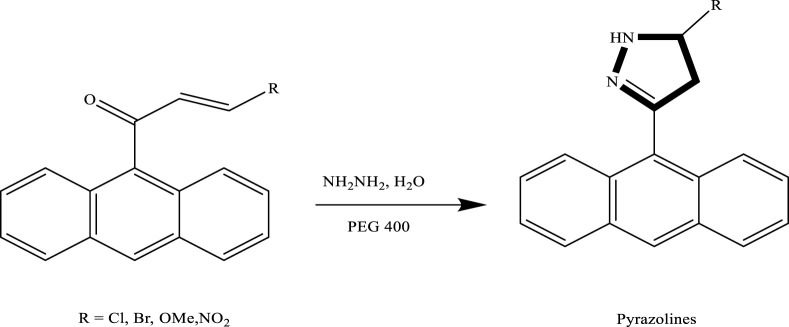
Green synthesis of 2-pyrazolines via the condensation of chalcone derivatives with hydrazine hydrate in PEG-400 as the reaction medium.

Yedukondalu et al. successfully synthesized substituted tetrahydrocarbazoles using a green approach. The reaction involved heating phenylhydrazine hydrochloride or 4-piperidone hydrochloride with substituted cyclohexanones or piperidone in polyethylene glycol (PEG) as the reaction medium. As shown in Scheme 3 this method enabled the efficient formation of various tetrahydrocarbazole derivatives under mild and environmentally friendly conditions (Vlocskó et al., 2023). Similarly, Lavania et al., successfully synthesized 2-pyrazolines through the condensation of chalcones with hydrazine hydrate in polyethylene glycol (PEG) as the reaction medium. This green protocol proved to be efficient, affording pyrazoline derivatives in good to excellent yields, which is given in Scheme 4.

### Formation of the pyrazole ring reactions in bio-based solvents

**SCHEME 5:**
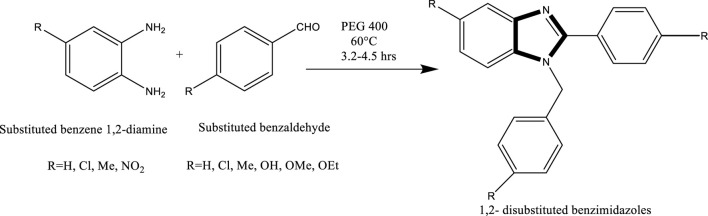
Synthesis of 1,2-disubstituted benzimidazoles via the condensation of substituted benzene-1,2-diamines with substituted benzaldehydes in PEG-400 at 60°C for 3.2–4.5 h.

Bhat et al. successfully synthesized 2-pyrazoline derivatives using a green reaction protocol catalysed by cerium chloride heptahydrate (CeCl_3_·7H_2_O) in ethyl lactate as the solvent. The method involved the condensation of chalcones with phenylhydrazine to afford 1,3,5-triaryl-2-pyrazolines in good yields. This approach highlights the effectiveness of combining a mild Lewis acid catalyst with a bio-based solvent for sustainable heterocycle synthesis (Vlocskó et al., 2023) (Scheme 5).

## Formation of the imidazole ring by reactions using PEG

**SCHEME 6:**
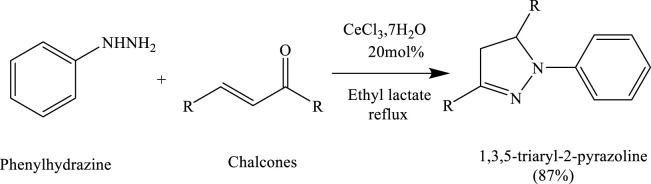
General mechanism for the PEG-400 mediated synthesis of 1,2-disubstituted benzimidazoles.

Mekala et al. developed an efficient method for the synthesis of 1,2-disubstituted benzimidazoles. The reaction proceeds through a nucleophilic attack by phenylenediamine on the carbonyl carbon of substituted benzaldehydes. The electrophilicity of the carbonyl carbon is significantly enhanced in the presence of PEG-400, making it a superior reaction medium compared to conventional solvents. Moreover, PEG-400 facilitates the removal of water generated during the condensation, as it readily dissolves in the medium. This promotes forward reaction kinetics and leads to high yields of the desired benzimidazole products under mild, green conditions (Vlocskó et al., 2023) (Scheme 6).

## Formation of the imidazole rings by reactions in bio-solvent

**SCHEME 7:**
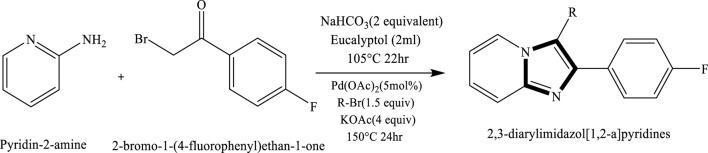
Synthesis of 2,3-diarylindazole-fused [1,2-a]pyridines via the reaction of pyridin-2-amine with 2-bromo-1-(4-fluorophenyl)ethan-1-one under basic conditions.

In groundbreaking study, Berteina-Rabinol et al. presented compelling evidence that eucalyptol possesses the potential to serve as an organic solvent in synthesis processes. This discovery highlights the possibility of utilizing eucalyptol as a viable alternative to traditional solvents in the one-pot synthesis of 2,3-diarylimidazol[1,2-a]pyridines. The synthesis involves a condensation reaction between 2-aminopyridine and bromoacetophenones, followed by a C-H activation at C-3. Notably, this solvent, derived from biomass and possessing recyclable properties, also exhibited remarkable efficacy in facilitating diverse transformations of heteroatom-containing heterocycles, encompassing oxygen, sulphur, and nitrogen (Vlocskó et al., 2023) (Scheme 7).

## Green synthesis of Schiff bases by using natural acid catalysts

Schiff bases, also known as imines, are compounds characterized by the presence of an azomethine group (–HC=N–) and are generally represented by the formula R_2_R_3_C=NR_1_. These compounds are typically formed through the condensation of primary amines with aldehydes or ketones. The first synthesis of Schiff bases was reported by Hugo Schiff in 1864. The classical method developed by Schiff involves the condensation of a primary amine with a carbonyl compound, with the concurrent removal of water—often achieved through azeotropic distillation—to drive the reaction to completion (Gundlewad, 2022).

## Synthesis of Schiff bases with grapes juice sweet lemon juice and aq. extract of unripe mango under solvent free condition by stirring methods

**SCHEME 8:**
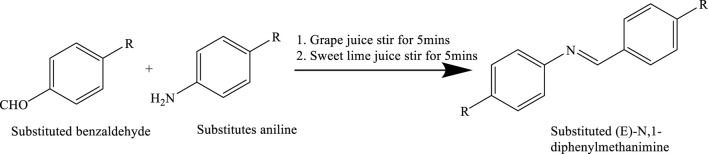
Green synthesis of Schiff bases through the condensation of substituted benzaldehydes with substituted anilines using natural acid catalysts such as grape juice and sweet lime juice.

Equimolar quantities of benzaldehyde (0.1 mol) and aniline (0.1 mol) were measured into separate beakers. Natural acid catalysts—namely grape juice, sweet lemon juice, and an aqueous extract of mango—were added in varying volumes (0.5 mL, 1.0 mL, 1.5 mL, 2.0 mL, and 2.5 mL) to the reaction mixtures. Each mixture was allowed to stand for 5–10 min, followed by stirring at room temperature for 2–4 min. Upon completion of the reaction, a pale-yellow solid product formed, which was collected, washed with distilled water, and purified via recrystallization using a minimal amount of ethanol. The same procedure was repeated using the other natural acid catalysts. The melting points of the products were determined by the open capillary method. Thin-layer chromatography (TLC) was employed for monitoring reaction progress and verifying purity, while product identification and confirmation of purity were further validated using mass spectrometry (Gundlewad, 2022) (Scheme 8).

## Natural catalysis: pineapple juice as an efficient catalyst for the Biginelli reaction

**SCHEME 9:**
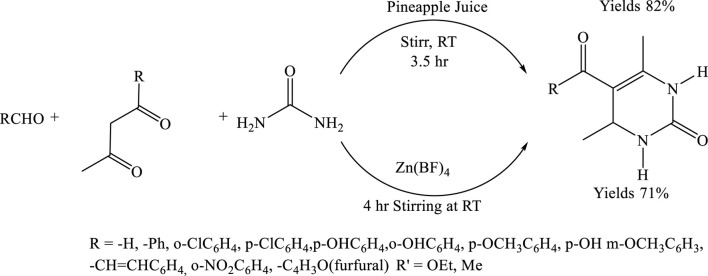
Green synthesis of 5-ethoxycarbonyl-6-methyl-4-aryl-3,4-dihydropyrimidin-2(1H)-ones via a three-component Biginelli reaction between substituted aldehydes (RCHO), β-keto esters, urea using pineapple juice as a natural catalyst at room temperature for 3.5 h.

To synthesize 5-ethoxycarbonyl-6-methyl-4-(4-methoxyphenyl)-3,4-dihydropyrimidin −2(1H)-one, equimolar amounts of p-methoxy benzaldehyde (1.36 g, 10 mmol), ethyl acetoacetate (1.30 g, 10 mmol), and urea (0.60 g, 10 mmol) were mixed in 1 mL of pineapple juice, serving as a natural acid catalyst. The reaction mixture was stirred at room temperature for 3.5 h, and the progress was monitored by thin-layer chromatography (TLC). Upon completion, the reaction mixture was filtered, and the resulting solid was washed with a small amount of distilled water. The crude yellow product was purified by recrystallization from ethanol, affording fine yellow crystals of the desired compound (Gundlewad, 2022) (Scheme 9).

### Synthesis of dihydropyridine

## Green synthesis of thiazolo- and benzothiazolo [3,2-a]pyrimidine derivatives using onion peel as a natural catalyst

**SCHEME 10:**
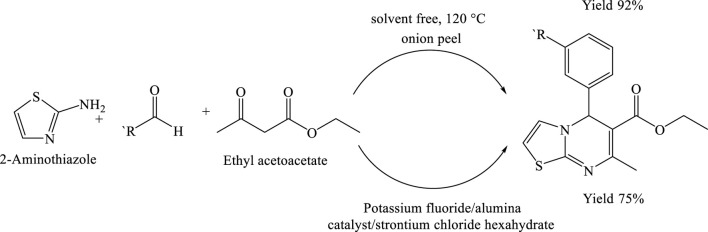
Solvent-free synthesis of 4H-thiazolo[3,2-a]pyrimidin-5-ones via the multi component reaction of 2-aminothiazole, substituted aldehydes, and ethyl acetoacetate at 120°C using onion peel powder as a natural catalyst.

In a solvent-free reaction system, a 50 mL round-bottom flask was charged with a mixture of powdered onion peel, 2-aminobenzothiazole (or 2-aminothiazole) (1.00 mmol), benzaldehyde (1.00 mmol), and ethyl acetoacetate (1.00 mmol). The contents were magnetically stirred to ensure homogeneous mixing, after which the reaction mixture was gradually heated to 120°C. The use of onion peel as a natural catalyst significantly enhanced the reaction efficiency, facilitating the synthesis of the target heterocyclic product. The reaction proceeded smoothly under these conditions, affording the desired product in an excellent yield of 91% within just 60 min. This result highlights the potential of onion peel as a sustainable, eco-friendly catalyst in green organic synthesis (Tan et al., n.d.) (Scheme 10).

## Asymmetric production of (S)-1-(benzofuran-2-yl)ethanol by *Lactobacillus paracasei*

**SCHEME 11:**
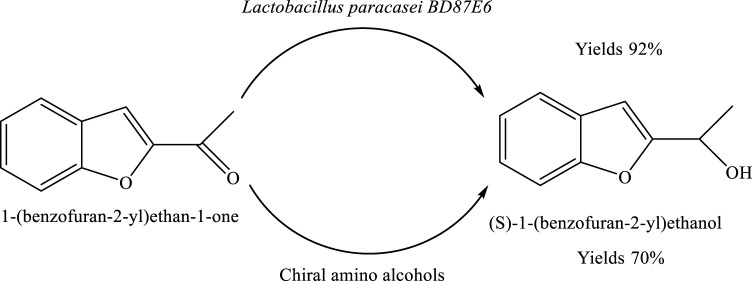
Bioreduction of 1-(benzofuran-2-yl)ethan-1-one to enantiomerically pure (S)-1-(benzofuran-2-yl)ethanol using *Lactobacillus* paracasei BD5YE6 in the presence of chiral amino alcohols.

The compound (S)-1-(benzofuran-2-yl)ethanol can be synthesized from 1-(benzofuran-2-yl)ethan-1-one through an optimized chemical reduction process. Under the established reaction conditions, the transformation proceeds with high efficiency, yielding the desired chiral alcohol in approximately 92% yield. This indicates a highly effective conversion of the starting ketone to the target product, highlighting the practicality of the method for preparative synthesis (Anderson et al., 2018) (Scheme 11).

## Microbial transformation of polydatin to resveratrol

**SCHEME 12:**
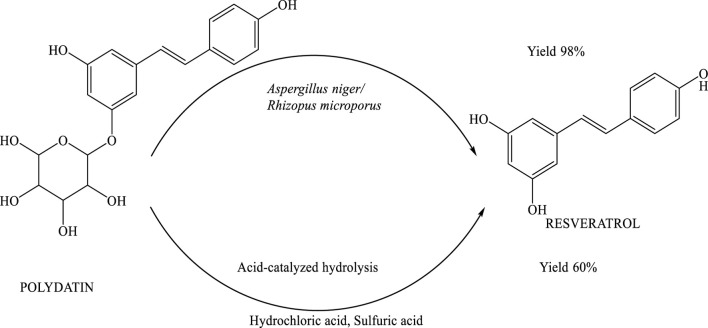
Biotransformation and acid-catalyzed hydrolysis of polydatin to resveratrol. The enzymatic pathway involves cellulase-producing fungi (Aspergillus niger and Rhizopus microporus), yielding resveratrol with 98% efficiency.

Polydatin was efficiently converted into resveratrol through fungal biotransformation using Aspergillus niger and Rhizopus microporus. These fungi played a pivotal role in catalyzing the hydrolysis process, enabling the selective removal of the glycosidic moiety from polydatin. The method demonstrated high efficiency, achieving a 98% yield of resveratrol, thereby highlighting the potential of this biocatalytic approach as a reliable and scalable strategy for natural product synthesis (Jeandet et al., 2020). The biotransformation of polydatin into resveratrol is mediated by a specific enzymatic process involving the cleavage of the β-glycosidic bond. This reaction is catalyzed by cellulase, an enzyme produced by Aspergillus niger. The enzyme effectively hydrolyzes the glycosidic linkage in polydatin, resulting in the formation of resveratrol—a compound known for its diverse biological and pharmacological activities. This enzymatic conversion represents a key step in the metabolic pathway, demonstrating the efficiency of Aspergillus niger in producing resveratrol from its natural precursor (Jeandet et al., 2020) (Scheme 12).

## Synthesis of coumarin derivatives via knoevenagel condensation

**SCHEME 13:**
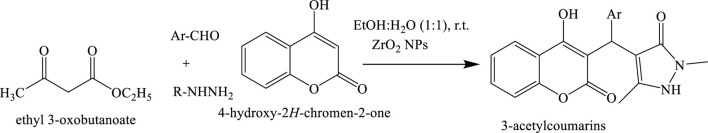
Synthesis of 3-acetylcoumarins via a multi-component reaction involving ethyl acetoacetate, aryl aldehydes (Ar–CHO), and substituted hydrazines (R–NHNH_2_). The reaction proceeds through the intermediate formation of 4-hydroxy-2H-chromen-2-one in an EtOH–H_2_O (1:1) solvent system under room temperature conditions, catalyzed by ZnO nanoparticles (ZnO NPs).

3-Acetylcoumarins were synthesized through a reaction between salicylaldehyde and ethyl acetoacetate, catalyzed by L-proline under environmentally friendly conditions. Triethanolamine was employed as the reaction medium, providing an efficient and green solvent system. The use of L-proline as a biodegradable organocatalyst underscores the green chemistry approach, facilitating the formation of 3-acetylcoumarins in a sustainable manner (Molnar et al., 2020) (Scheme 13).

## Photocatalytic synthesis as a green chemistry

The conventional production of 4-tert-butylbenzaldehyde, an important intermediate in fine chemical synthesis, involves the stoichiometric oxidation of 4-tert-butyltoluene (4-TBT) using permanganate as the oxidizing agent. However, this method generates large quantities of organic and inorganic waste, contributing significantly to environmental pollution and raising concerns about its sustainability (Herrmann et al., 2007).

$$4‐\text{tert}–C_{4}H_{9}–C_{6}H_{4}–{\text{CH}}_{3}+4{\text{KMnO}}_{4}+6H_{2}{\text{SO}}_{4}\;\to \;4‐\text{tert}–C_{4}H_{9}–C_{6}H_{4}–\text{CHO}+2K_{2}{\text{SO}}_{4}+4{\text{MnSO}}_{4}+11H_{2}O$$

In contrast, the oxidation of 4-tert-butyltoluene (4-TBT) demonstrates complete selectivity in both gas and liquid phases, yielding 4-tert-butylbenzaldehyde as the sole product. This high selectivity is achieved by irradiating titania with near-UV light under ambient air and room temperature conditions, without the use of additional catalysts or reagents. The process underscores the efficiency and precision of this photocatalytic oxidation under mild, environmentally benign conditions (Herrmann et al., 2007).

$$4‐\text{tert}–C_{4}H_{9}–C_{6}H_{4}{\text{CH}}_{3}+O_{2}\;\to \;4‐\text{tert}–C_{4}H_{9}–C_{6}H_{4}–\text{CHO}+H_{2}$$

## Water as a green chemistry solvent

### An eco-friendly and sustainable synthesis of pyrimido[4,5-d]pyrimidines

A novel and environmentally friendly method for synthesizing pyrimido[4,5-d]pyrimidines has been developed through a one-pot reaction of barbituric acid, an aldehyde, and urea or thiourea in aqueous medium. This solvent-free and catalyst-free approach avoids the use of hazardous organic solvents, corrosive acids or bases, and toxic reagents. The reaction proceeds efficiently in water—a renewable and green solvent—affording water-insoluble solid products with excellent purity and high yields within a short reaction time. This protocol exemplifies the principles of Green Chemistry by minimizing waste, eliminating toxic inputs, and employing a sustainable reaction medium (Sánchez Morales et al., 2024).

**SCHEME 14:**
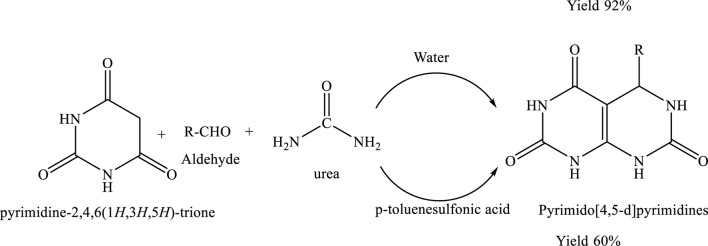
Green synthesis of pyrimido[4,5-d]pyrimidines via a three-component reaction involving pyrimidine-2,4,6(1H,3H,5H)-trione, an aromatic aldehyde (R–CHO), urea in water.

The green chemistry reaction afforded a 92% yield, indicating high efficiency and effective conversion of reactants to the desired product. This excellent yield highlights the suitability of the reaction conditions and supports the sustainability of the methodology. The result reflects the core principles of green chemistry, including waste minimization and efficient resource utilization, thereby contributing to the development of more sustainable chemical processes (Kidwai et al., 2007) (Scheme 14).

### Synthesis of 2-aminothiazoles by green solvent water

**SCHEME 15:**
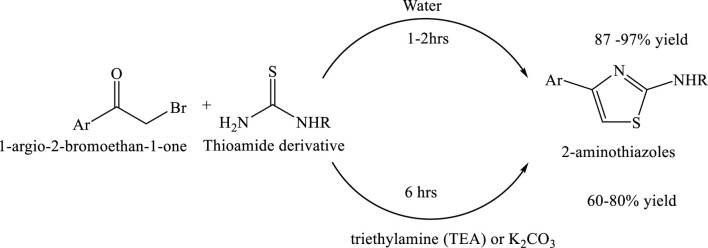
Green synthesis of substituted 2-aminothiazoles via the reaction of 1-aryl-2-bromoethan-1-one with thioamide derivatives. The reaction proceeds efficiently in water at ambient temperature within 1–2 h, yielding 2-aminothiazoles in 87%–97% yield without the need for catalysts.

A sustainable and environmentally benign method has been developed for the synthesis of substituted 2-aminothiazoles using water as the sole solvent. This catalyst-free and co-solvent-free reaction proceeds efficiently at ambient temperature, supporting energy conservation and reducing environmental impact. The method affords excellent isolated yields and adheres to the principles of Green Chemistry by minimizing waste and avoiding hazardous reagents. Notably, this protocol has been successfully applied to the synthesis of the anti-inflammatory drug fanetizole, underscoring its potential for eco-friendly pharmaceutical production (Potewar et al., 2008) (Scheme 15).

## Microwave-assisted green synthesis

### Synthesis of quinoline derivatives

**SCHEME 16:**
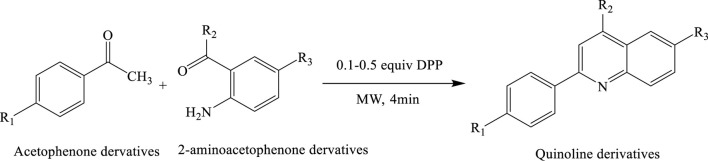
Solvent-free synthesis of quinoline derivatives via Friedländer condensation of acetophenone derivatives and 2-aminoacetophenone derivatives using diphenyl phosphate (0.1–0.5 equiv.) as a catalyst under microwave irradiation.

A mini-library of 12 quinoline derivatives was synthesized via a solvent-free Friedländer condensation between acetophenone and 2-aminoacetophenone, catalysed by diphenyl phosphate (0.1–0.5 equiv.). The reaction was carried out under microwave irradiation and completed within just 4 min, offering a highly efficient and sustainable approach. This method significantly improved product yields—up to 85%—compared to only 24% under conventional heating. The protocol exemplifies the principles of green chemistry by eliminating hazardous solvents, minimizing energy consumption, reducing waste, and enhancing overall reaction efficiency (De La Hoz and Prieto, 2016; Gohain et al., 2004) (Scheme 16).

### Microwave-assisted cycloaddition between isoprene and ethyl glyoxylate adsorbed on graphite

**SCHEME 17:**
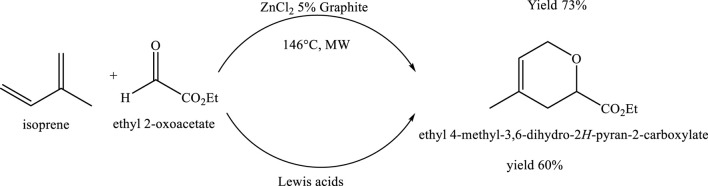
Microwave-assisted cycloaddition of isoprene and ethyl glyoxylate (ethyl 2-oxoacetate) in the presence of ZnCl_2_ and 5% graphite as a catalytic system.

The cycloaddition reaction between isoprene and ethyl glyoxylate serves as an efficient and sustainable example of green chemistry. Conducted under microwave irradiation, the reaction reaches completion in just 10 min (10 cycles of 1 min each) at a final temperature of 146°C. This approach significantly accelerates the reaction rate while reducing overall energy consumption compared to conventional heating. The use of microwave energy enables precise control over reaction parameters, resulting in a 73% yield with minimal by-product formation. The combination of rapid reaction time, high selectivity, and reduced waste underscores the environmental benefits of this method, aligning well with the core principles of green chemistry (De La Hoz and Prieto, 2016) (Scheme 17).

## Conclusion

This review underscores the significant advancements in green chemistry approaches for organic synthesis. The incorporation of sustainable strategies—such as the use of ionic liquids, bio-based solvents, natural acid catalysts, and microwave-assisted techniques—has greatly reduced the environmental footprint of chemical transformations. These methodologies not only improve atom and energy efficiency but also minimize waste generation and eliminate the need for hazardous reagents.

The examples presented demonstrate the strong potential of green chemistry to revolutionize pharmaceutical and fine chemical synthesis by offering safer, more efficient, and economically viable alternatives. Notably, high product yields, reduced reaction times, and simplified protocols emphasize the scalability and industrial relevance of these green processes. Continued innovation and research in this field are essential for advancing sustainable practices in chemical manufacturing. By embracing green chemistry principles, the scientific community can contribute meaningfully to environmental protection and the development of a more sustainable future for the chemical industry.
